# Optimized Method of Extracting Rice Chloroplast DNA for High-Quality Plastome Resequencing and *de Novo* Assembly

**DOI:** 10.3389/fpls.2018.00266

**Published:** 2018-02-28

**Authors:** Takeshi Takamatsu, Marouane Baslam, Takuya Inomata, Kazusato Oikawa, Kimiko Itoh, Takayuki Ohnishi, Tetsu Kinoshita, Toshiaki Mitsui

**Affiliations:** ^1^Department of Life and Food Sciences, Graduate School of Science and Technology, Niigata University, Niigata, Japan; ^2^Laboratory of Biochemistry, Faculty of Agriculture, Niigata University, Niigata, Japan; ^3^Center for Education and Research of Community Collaboration, Utsunomiya University, Utsunomiya, Japan; ^4^Kihara Institute for Biological Research, Yokohama City University, Yokohama, Japan

**Keywords:** *de novo* assembly, chloroplast DNA, next-generation sequencing, plastid genome, NUPTs, MTPTs, *Oryza sativa*

## Abstract

Chloroplasts, which perform photosynthesis, are one of the most important organelles in green plants and algae. Chloroplasts maintain an independent genome that includes important genes encoding their photosynthetic machinery and various housekeeping functions. Owing to its non-recombinant nature, low mutation rates, and uniparental inheritance, the chloroplast genome (plastome) can give insights into plant evolution and ecology and in the development of biotechnological and breeding applications. However, efficient methods to obtain high-quality chloroplast DNA (cpDNA) are currently not available, impeding powerful sequencing and further functional genomics research. To investigate effects on rice chloroplast genome quality, we compared cpDNA extraction by three extraction protocols: liquid nitrogen coupled with sucrose density gradient centrifugation, high-salt buffer, and Percoll gradient centrifugation. The liquid nitrogen–sucrose gradient method gave a high yield of high-quality cpDNA with reliable purity. The cpDNA isolated by this technique was evaluated, resequenced, and assembled *de novo* to build a robust framework for genomic and genetic studies. Comparison of this high-purity cpDNA with total DNAs revealed the read coverage of the sequenced regions; next-generation sequencing data showed that the high-quality cpDNA eliminated noise derived from contamination by nuclear and mitochondrial DNA, which frequently occurs in total DNA. The assembly process produced highly accurate, long contigs. We summarize the extent to which this improved method of isolating cpDNA from rice can provide practical progress in overcoming challenges related to chloroplast genomes and in further exploring the development of new sequencing technologies.

## Introduction

Chloroplasts, which are important cellular organelles that provide energy to plants, have an independent, circular, double-stranded DNA. The chloroplast genome (plastome), which ranges in size from 110 to 200 kb, consists of a pair of IRs, a LSC, and a SSC. The first whole-chloroplast-genome sequencing of rice (Hirai et al., 1985; Hiratsuka et al., 1989), Arabidopsis (Sato et al., 1999), and maize (Maier et al., 1995), based on Sanger technology, provided a basic understanding of the genome’s structure and function. NGS technology now offers the promise to further the development of chloroplast genome studies, and has accelerated analyses to the point where more than 1,600 accessions of complete chloroplast genome sequences from land plants are available in public databases^1^. The advance of high-throughput, high-resolution analyses has facilitated population genetics and evolutional studies focused on the chloroplast genome (Morris et al., 2011; Wu et al., 2015; Tong et al., 2016). Organelle genomes have less diversity and exert less influence on phenotype than the nuclear genome (Wolfe et al., 1987; Kahlau et al., 2006; Drouin et al., 2008), although some studies have shown that variation in organelle genomes can influence variation in phenotypes (Moison et al., 2010; Joseph et al., 2013; Tang et al., 2013). Joseph et al. (2013) demonstrated that the cytoplasmic genome plays a central role in controlling natural variations in metabolomic networks within a reciprocal Arabidopsis Kas × Tsu recombinant inbred line population. Roux et al. (2016) reported cyto-nuclear co-adaptation by creating a unique series of 56 cytokines resulting from cytoplasmic substitutions among eight natural Arabidopsis species. More recent widespread reports show that interactions between nuclear and cytoplasmic genomes shape natural variation (Greiner and Bock, 2013; Sloan, 2015; Roux et al., 2016). This factor gives rise to new opportunities for using organelle genomes as novel breeding targets (Maliga, 2001; Wang et al., 2008). The plastome thus presents an attractive target for genome engineering and a promising alternative to nuclear transformation (Olejniczak et al., 2016). Indeed, chloroplast genomic studies have crucial translational and biotechnological applications owing to the genome’s ability to express > 120 foreign genes from different organisms (Daniell et al., 2016). Accordingly, the efficient sequencing of plastid genomes, which requires highly purified plastid DNA, will allow the production of transplastomic plants (Diekmann et al., 2008).

Rice, one of the most important food crops in the world, has important syntenic relationships with the other cereal species and is a model for monocots and grasses. The chloroplast genome of *Oryza sativa* is 134,525 base pairs long, with 159 unique genes, including 38 tRNA, 8 rRNA, and 108 protein-coding genes (Hiratsuka et al., 1989). The evolutionary transfer of plastid DNA fragments to the nuclear and mitochondrial genomes is frequently found in plants (Lewin, 1984; Martin and Herrmann, 1998; Matsuo et al., 2005). Such transfers to the nuclear genome (nuclear plastid DNA, NUPTs) and to the mitochondrial genome (mitochondrial plastid-like sequences, MTPTs) are more abundant in rice than in other higher plants (Yoshida et al., 2014). As plastome sequencing is frequently based on total DNA, this might reduce the mapping accuracy owing to the difficulty in selecting plastid-derived reads from the whole-genome sequence, which includes NUPT- and MTPT-derived reads, obtained by short read sequencing. In addition, total cellular DNA contains only 1–10% cpDNA, and the amount decreases during plant development (Baumgartner et al., 1989; Oldenburg and Bendich, 1991; Shaver et al., 1995; Oldenburg and Bendich, 2004). These problems reduce the efficiency of sample multiplex analysis in a single sequencing run on low-output sequencers such as MiSeq, MiniSeq, and PacBio RS II. Therefore, the isolation of high-purity cpDNA will improve the accuracy and cost-efficiency of plastome sequencing. Four procedures for cpDNA isolation have been reported: Percoll density gradient centrifugation to obtain intact chloroplasts free of other organelles (Lang and Burger, 2007; Kaneko et al., 2016); high salt concentration to remove contaminating DNA ionically attached to the chloroplast surface (Shi et al., 2012); the use of DNase I to digest DNA bound to the chloroplast surface (Kolodner and Tewari, 1979); and liquid nitrogen pre-treatment to prevent nuclear breakage (Hirai et al., 1985).

Shi et al. (2012) reported that the DNase I method digested not only the contaminating DNA but also cpDNA within chloroplasts. So we compared Percoll density gradient centrifugation (PG), high salt (HS), and liquid nitrogen coupled with a sucrose gradient (LN) to optimize cpDNA analysis by NGS. We sequenced the highly purified cpDNA to compare the advantage of cpDNA sequencing with whole-genome sequencing. We also assessed the performance of SNP/insertion–deletion (indel) calling and *de novo* assembly on the plastome to evaluate the effect of cpDNA purity on NGS analysis.

## Materials and Methods

### Plant Material

We germinated seeds of several rice (*O. sativa*) cultivars: temperate japonica Nipponbare (Shiga Prefecture Agricultural Research Center) and Koshihikari (Niigata Agricultural Research Institute); tropical japonica Sensho, Urasan, Padi Perak, and Khao Nok (NARO, Genetic Resources Center); aus Chinsurah Boro 2 (Tohoku University) and Kasalath; and indica 93-11 (National Institute of Genetics).

### Protocols for Chloroplast Isolation

The three techniques selected for cpDNA isolation are described as follow: To determine the best method, we first performed three independent experiments to extract the cpDNA from bulk samples of Nipponbare by using each method (LN, HS, PG method). Then, the cpDNA from bulk samples of other cultivars were extracted once or twice by LN method.

#### Updated Liquid Nitrogen–Sucrose Density Gradient Centrifugation (LN) Method

**FIGURE 1:**
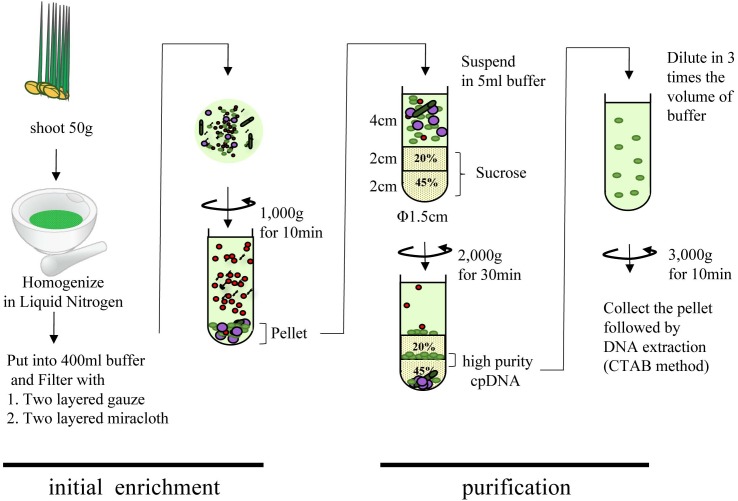
Flowchart of chloroplast DNA isolation using liquid nitrogen coupled with sucrose gradient centrifugation.

Rice seeds were sterilized and germinated on moist filter paper in culture dishes in the dark at 28°C for up to 4 days. The germinated seeds were dispersed onto a layer of water-agarose gel laid to block contamination by fungi over 1/2 MS medium agarose gel. Plants were placed in a growth chamber (28:23°C, 12:12 h, light:dark; 20,000 lux) for 8 days. cpDNA was isolated as described (Hirai et al., 1985) with some modifications (**Figure 1**). All procedures were performed at 4°C, and all centrifugations were performed in a CP80NX ultracentrifuge (Hitachi) with a P40ST swing rotor and 13PA tubes. In brief, 50 g of fresh shoot was cut into pieces (∼3 cm), frozen in liquid nitrogen for 3 min, and gently ground into a fine powder with a mortar and pestle. The material was suspended in 400 mL of isolation buffer (50 mM Tris-HCl pH 8.0, 0.35 M sucrose, 7 mM EDTA, 5 mM 2-mercaptoethanol, 0.1% BSA) and incubated for 5 min in the dark. The suspension was filtered through two layers of gauze and then two layers of Miracloth (Merck). The filtrate was centrifuged at 1000 × *g* for 10 min. The pellet was suspended in 5 mL of isolation buffer, and the suspension was loaded slowly onto a stepwise 20%/45% discontinuous sucrose density gradient in 50 mM Tris-HCl (pH 8.0), 0.3 M sorbitol, and 7 mM EDTA. The gradient was centrifuged at 2000 × *g* for 30 min in a swinging bucket rotor. The green band at the 20%/45% sucrose interface was collected, diluted with three volumes of isolation buffer, and centrifuged at 3000 × *g* for 10 min in a swinging bucket rotor. Genomic DNA was isolated from the pellet by the cetyltrimethylammonium bromide method (Shi et al., 2012) or with a DNeasy Plant Mini Kit (Qiagen).

#### Modified High Salt (HS) Method

Sterilized seeds were grown on 0.8% agar in the dark at 28°C for 7 days and then in natural daylight at 28°C for 14 days. cpDNA was isolated using the protocol described in Shi et al. (2012). All procedures were performed at 4°C. In brief, 20 g of fresh leaves was cut into small pieces (∼1 cm) and homogenized for 30 s in 400 mL of buffer A (1.25 M NaCl, 0.25 M ascorbic acid, 10 mM sodium metabisulfite, 12.5 mM borax, 50 mM Tris-HCl pH 8.0, 7 mM EDTA, 1% [w/v] PVP-40, 0.1% [w/v] BSA, and 1 mM DTT). The homogenate was filtered through two layers of Miracloth, and the filtrate was centrifuged at 200 × *g* for 20 min to remove starch granules, nuclei, tissue debris, and aggregates. The supernatant was centrifuged at 3500 × *g* for 20 min, and the pellet was suspended in 250 mL buffer B (1.25 M NaCl, 12.5 M borax, 1% [w/v] PVP-40, 50 mM Tris-HCl pH 8.0, 25 mM EDTA, 0.1% [w/v] BSA, and 1 mM DTT) to increase the purity of the isolated cpDNAs. This step was performed twice. cpDNA was extracted with a DNeasy Plant Mini Kit (Qiagen).

#### Percoll Gradient (PG) Centrifugation Method

Sterilized seeds were grown on 0.8% agar at 30°C in the dark for 11 days, and then under continuous light at 28°C for 3 days for greening. cpDNA was isolated as in our previous report (Kaneko et al., 2016). All procedures were performed at 4°C. In brief, rice shoots (20 g) were homogenized in 20 mL of isolation buffer (50 mM HEPES-KOH pH 7.5, 0.33 M sorbitol, 5 mM MgCl_2_, 5 mM MnCl_2_, 5 mM EDTA, and 50 mM sodium ascorbate). The homogenate was filtered through four layers of gauze and then four layers of Miracloth. The filtrate was layered onto a cushion of 80% (v/v) Percoll (Sigma) in the above isolation buffer (except for the sodium ascorbate) and centrifuged at 2000 × *g* for 4 min. The crude chloroplasts on the Percoll surface were collected and diluted with more than twice the volume of isolation buffer, and then layered onto a discontinuous density gradient of 40 and 80% Percoll. The gradient was centrifuged at 4000 × *g* for 10 min. Intact chloroplasts enriched around the 40%/80% Percoll interface were collected and centrifuged again as before. Intact chloroplasts were collected, diluted with five volumes of isolation buffer, and centrifuged at 2000 × *g* for 4 min. cpDNA was extracted with a DNeasy Plant Mini Kit (Qiagen).

In all three protocols, DNA were eluted from the DNeasy spin column using 80 μl (LN and PG method) or 40 μl (PG method) of elution buffer. Two microliters of the isolated cpDNA was loaded and separated in 0.8% agarose gel and visualized with ethidium bromide.

### Genome Copy Number Analysis by qPCR

We tested the cpDNA purity of DNA isolates by quantifying the number of copies of chloroplast, mitochondrial, and nuclear genomes. cpDNA, mtDNA, and ncDNA were quantified by quantitative real-time PCR (qPCR) using SsoFast EvaGreen Supermix (Bio-Rad) on a CFX96 real-time PCR system/C1000 Thermal Cycler (Bio-Rad). The thermocycling conditions were denaturation at 98°C for 2 min, and 39 cycles of 98°C for 2 s and 60°C for 5 s. To analyze genome copy number, we designed two sets of primers for each genome (Supplementary Table 1) to improve the accuracy of quantification, and used the mean as the threshold cycle (Ct) value of each genome. First, we calculated the PCR amplification efficiencies of each primer pair with a dilution series of a plasmid standard (10^4^ to 10^9^ copies of pGEM-T::Actin1::GAPDH::atpI::psbA::cob::coxII). The amplification efficiencies of all six genes were close to 2.0 (1.93–2.03), and *R^2^* values were between 0.989 and 0.999. Next, we calculated the copy ratios of cpDNA/ncDNA and cpDNA/mtDNA from the standard curves drawn from the above dilutions and compared these results of absolute quantification with the results of relative quantification using the 2^-ΔΔct^ method (Schmittgen and Livak, 2008). Since the results of relative quantification were almost the same as those of absolute quantification, we used the relative quantification method in the subsequent qPCR analysis, giving priority to efficiency. Samples were assayed in at least three technical replicates, and the average copy ratios were calculated. Then, each genome DNA content in the extracted DNA was estimated from the copy ratio and genome size (plastid, 134,525 bp; mitochondrial, 490,520 bp; nuclear, 373,245,519 bp).

### DNA Library Construction and Next-Generation Sequencing

We used 1 ng of Nipponbare purified cpDNA as input for the Nextera XT DNA library preparation kit and the Nextera XT index kit (Illumina). Constructed libraries were sequenced on an Illumina MiSeq sequencer (300 bp paired-end) with the MiSeq reagent kit v3 (Illumina). All procedures followed the manufacturer’s instructions.

### Sequence Mapping and Variant Detection

The sequenced paired-end reads from the purified cpDNA and three sets of whole-genome sequencing reads (total DNA [tDNA] 1–3) downloaded from public databases were trimmed in Trimmomatic v. 0.33 software (Bolger et al., 2014) with the following parameters: SLIDINGWINDOW: 8:20; TRAILING: 20; MINLEN: 90 (tDNA 1), 100 (purified cpDNA and tDNA 2), 76 (tDNA 3). The processed reads were aligned to the rice plastid reference genome (X15901.1) or a combined plastid (X15901.1)-mitochondrial (BA000029) reference genome by using the BWA-MEM v. 0.7.15 algorithm (Li and Durbin, 2009) with default parameters. PCR duplicates in BAM files were marked with Picard tools v. 1.68 software^2^. Then local realignment of reads around indels was done in GATK (Genome Analysis Toolkit) IndelRealigner software (DePristo et al., 2011). To estimate cpDNA purity, we extracted unaligned reads from BAM files in SAMtools software (Li et al., 2009) and re-aligned them on the rice mitochondrial reference genome (BA000029). These unmapped hits were extracted again and realigned on the rice nuclear reference genome (IRGSP-1.0). The coverage depth of each genome was calculated from the number and length of high-quality reads in a 250-nt sliding window. To calculate allele frequency at individual plastid genome positions, we generated wig files describing the base (A/C/G/T) content in a 1-nt sliding window from BAM files in igvtools v. 2.2 software (Robinson et al., 2011; Thorvaldsdóttir et al., 2013). After removal of data neighboring indels because of low reliability, we calculated first and second allele frequencies and coverage depths from wig files with a custom Perl script and then visualized them in 3D scatter plots using the scatterplot3d v. 0.3-37 tool of R (Ligges and Mächler, 2003). For variant calling, we used the SAMtools mpileup v. 1.4.1 tool (Li et al., 2009) with default parameters and GATK HaplotypeCaller v. 3.6 software (DePristo et al., 2011) with the ‘-ploidy 1’ parameter to compare SNPs and small indels from BAM files. We filtered out heterozygous and low-quality variants (QUAL < 20) in SAMtools, and low-quality variants (QUAL < 20) in GATK.

### *De Novo* Assembly

PCR duplicates were removed from paired-end reads using the *k*-mer-based method implemented in a Perl script^3^. From paired-reads of total DNA in BAM files, aligned plastid genome reads were extracted for enrichment of plastid reads, and then PCR duplicate reads were filtered out. Contigs were assembled from these reads in SOAP-denovo2 software (Luo et al., 2012) with various sets of *k*-mer parameters (Supplementary Table 4). After assembled scaffolds shorter than 500 bp were filtered out, sequences were compared against the plastid reference genome by NCBI BLAST 2 (Tatusova and Madden, 1999). Alignment results and detected SNPs/indels were visualized by Circos software (Krzywinski et al., 2009).

## Results and Discussion

### Comparison of Chloroplast DNA Isolation Methods for NGS Sequencing

Many areas of chloroplast research require assessment of the quality and quantity of cpDNA. Although assessment several methods have been developed, there is little discussion in the literature of whether they provide similar quality of information (Rowan et al., 2009; Rowan and Bendich, 2011). Large-scale studies of genome variation and evolution, which rely on large quantities of plant material, also require cpDNA isolation. Here, we compared three methods of isolating cpDNA (Supplementary Table 2): updated liquid nitrogen–sucrose density gradient centrifugation (LN), high-salt buffer (HS), and Percoll gradient centrifugation (PG). The original LN protocol was improved at various steps (Hirai et al., 1985) associated with gradient centrifugation to isolate cpDNA (**Figure 1**). The main improvements, made to allow the use of general laboratory instruments and to simplify the method, were the use of a mortar and pestle instead of a liquid nitrogen–resistant mixer, and omission of 65% sucrose following a preliminary experiment that showed heavy contamination of nuclear and mitochondrial DNA at the 45%/65% interface. The protocol was further extended to the isolation of cpDNA from several rice accessions from four cultivar groups.

**FIGURE 2:**
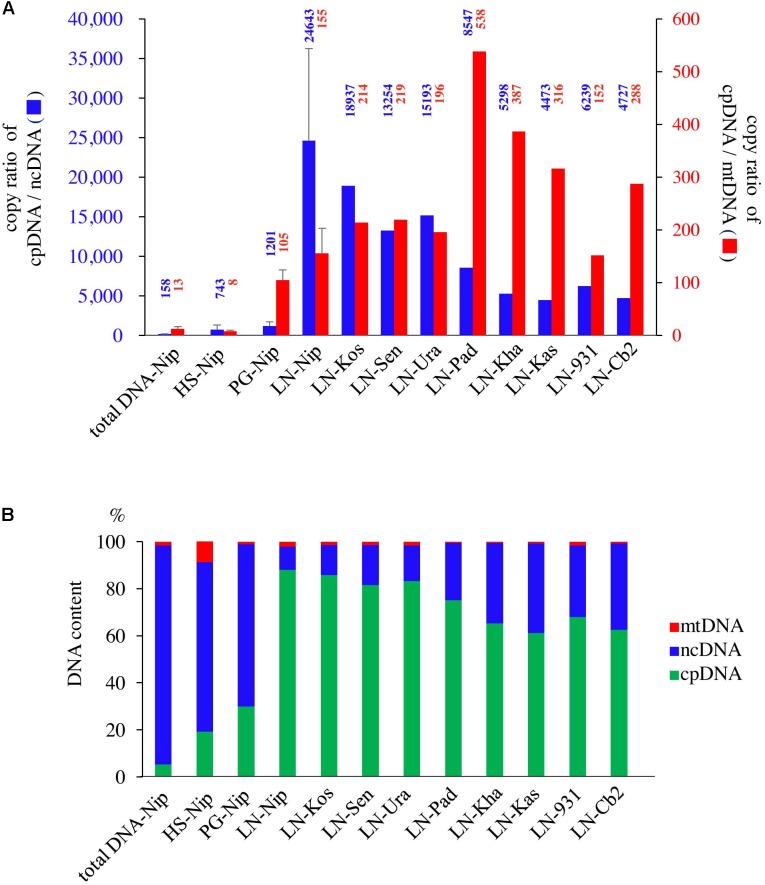
Evaluation of three cpDNA isolation methods. qPCR results of extracted cpDNA: **(A)** copy number ratio; **(B)** genomic DNA component rate. HS, high salt; PG, Percoll gradient centrifugation; LN, liquid nitrogen–sucrose gradient centrifugation. Nip, Nipponbare; Kos, Koshihikari; Sen, Sensho; Ura, Urasan; Pad, Padi Perak; Kha, Khao Nok; Kas, Kasalath; 931, *indica* 93-11; Cb2, Chinsurah Boro 2.

qPCR was used to determine the purity of the cpDNA samples for NGS. The HS method enriched not only cpDNA but also mtDNA (**Figure 2**), which may reduce cpDNA purity. The PG method enriched cpDNA with minimal contamination of mtDNA, but still with significant amounts of ncDNA. On the other hand, the LN technique consistently gave high purity, with cpDNA copy number ratios of 24 643× ncDNA and 155× mtDNA **(Figure 2A**, LN-Nip). A high ratio is critical to reducing misaligned reads on the plastome, such as NUPTs or MTPTs, and to increasing NGS accuracy. Moreover, cpDNA accounted for up to 88% of the total isolated DNA (**Figure 2B**), which is likely to provide meaningful cost-effectiveness of an NGS run. The LN method yielded high-purity cpDNA in all nine cultivars tested. These results show the potential for the LN method to be applied to a wide range of rice cultivars, as we have since confirmed (unpublished data).

**FIGURE 3:**
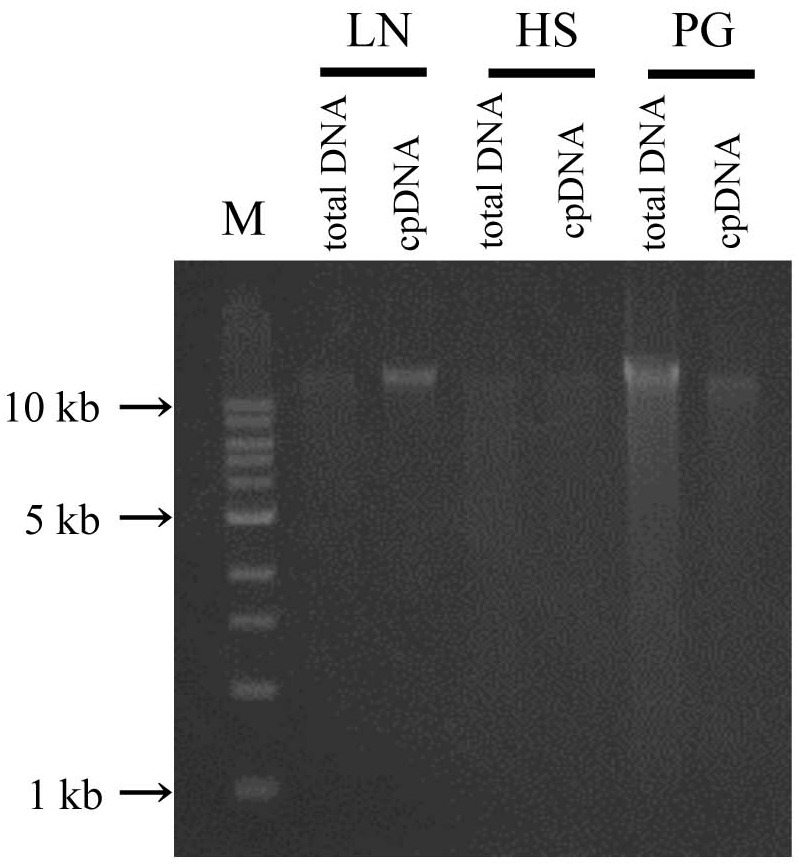
Chloroplast DNA visualization in agarose gel. Total DNA and cpDNA prepared by LN, HS, and PG protocols were subjected to 0.8% TAE agarose gel electrophoresis. M represents DNA size markers.

In the updated LN method, isolated DNA gave a well defined electrophoresis band, which is indicative of undegraded DNA (**Figure 3** and Supplementary Table 2). This DNA could be of sufficient quality not only for short-read sequencing, but also for mate-pair and long-read sequencing. The high-quality pure cpDNA isolated from LN method would be suitable to facilitate the plastome sequencing using the novel third-generation DNA sequencing technologies such as PacBio RS II and Oxford Nanopore MinION. This process may overcome the hard-to-assembly IRs regions composed of more than 20,000 base pairs long, and can enable new insights such as large indels and structural variations. A large amount of chloroplast pellet was collected, from which abundant cpDNA was extracted: 800 ng of cpDNA from 50 g of shoot. We also confirmed that as little as 4 g of shoot gave enough cpDNA for our NGS platform (Illumina MiSeq coupled with Nextera XT DNA library preparation). However, the DNA obtained from the HS and PS protocols displayed very weak bands and smeared, indicative of low DNA yield (**Figure 3** and Supplementary Table 2).

### Assessments of Resequencing: Mapping and Variant Calling

**FIGURE 4:**
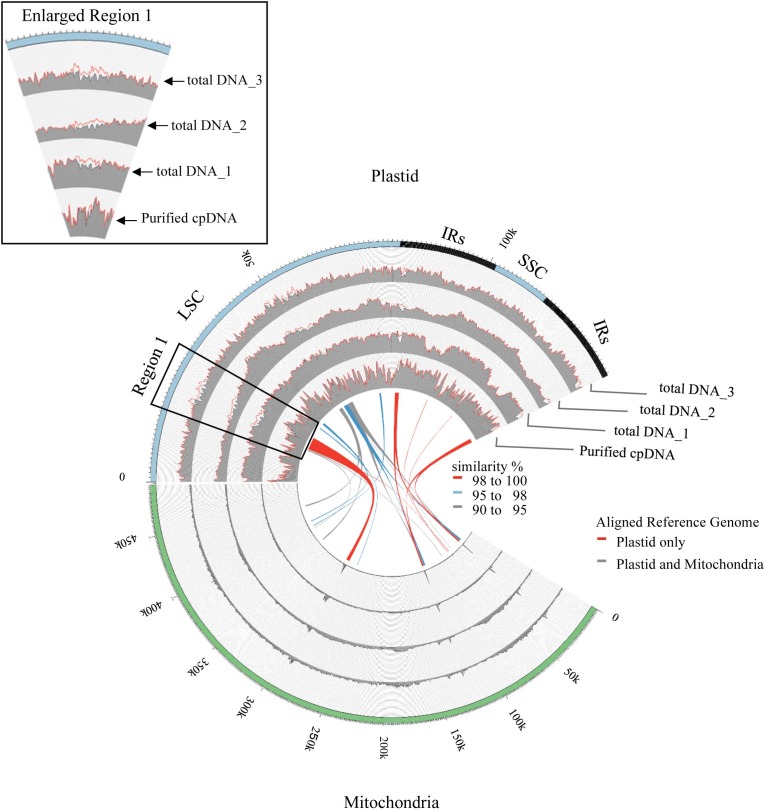
Visualization of read alignment on plastid reference genome. FASTQ reads were aligned on plastid reference genome only (red line) or combined plastid–mitochondrial reference genomes (gray area) followed by visualization of coverage depth in a 250-nt sliding window. Outermost circle shows common plastid genome structures: LSC, large single-copy; SSC, small single-copy; IRs, pair of inverted repeats. Inner colored ribbons mark BLASTN-identified PTMTs; width represents homology; color represents similarity (%).

Illumina sequencing produced 4,105,152 paired-end reads with an average read length of 175 bp and a total of 718,401,600 bases (**Table 1**). We downloaded three sets of whole-genome sequence data (tDNA 1–3) from the public database to assess the influence of cpDNA purity on NGS analysis (**Table 1**). The sequence reads were aligned to the Nipponbare plastid reference genome (X15901.1) to discover putative SNPs and small indels. Over 78% of reads were aligned in the cpDNA purified by the LN method, versus only 1.2–10.2% in the tDNA (**Table 1**), corresponding to ratios of 7.7× to 66× tDNA. The plastid genome (pt) was highly enriched in the cpDNA compared with those in three tDNAs (**Table 2**). Massively parallel sequencing gave an increase of at least fivefold in cpDNA purity compared with leaf tDNA (range, <3–30%) (Nock et al., 2011). The LN protocol is thus a feasible replacement for the PG and HS methods. The HS method isolated only pea cpDNA (Bookjans et al., 1984), and its improvement (Shi et al., 2012) did not improve purity. Hirao et al. (2008) considered the use of sucrose density gradient centrifugation as the best method for separating ncDNA contamination from cpDNA, which qPCR results (**Figure 2**) and the Illumina sequencing (**Table 2**) strongly supported, indicating high enough yield and purity to perform subsequent resequencing and genome assembly. The small difference between the results of mapping and qPCR analysis is likely due to the calculation methods, since qPCR calculates copy ratio from two target regions on each genome, whereas NGS aligns sequences across the whole genome. Visualization of the read alignment shows that the plastid reference genome is sufficiently covered in each sample (**Figure 4**). While tDNA samples show uniform coverage depth across the reference genome, the purified cpDNA shows irregular depth. The inconsistency would arise from the properties of the Nextera XT library kit, since a high number of PCR enrichment cycles can easily cause PCR-dependent coverage bias (Lan et al., 2015).

**Table 1:** Summary of NGS samples and aligned results.

	Library			Reads (After_QC)			Aligned reads		
DNA	SRA	Instrument	Layout	Read1	Read2	# Reads	# pt	# pt_uniq	% pt
Purified cpDNA	This study	Miseq	Paired	204	146	4,105,152	3,233,842	3,230,265	78.8
Total DNA_1	SRR1239746	Hiseq2000	Paired	90	89	10,595,500	1,076,775	1,003,940	10.2
Total DNA_2	SRR1614244	Hiseq2000	Paired	101	101	70,075,112	927,471	898,850	1.3
Total DNA_3	SRR077421	GAII	Paired	76	76	55,180,822	673,954	648,676	1.2
	SRR077422								
	SRR077425								

*Purified cpDNA: Nipponbare cpDNA obtained by LN method (**Figures 2A,B**, column 4). Total DNAs: published Nipponbare whole-genome sequencing reads downloaded from public database. Reads 1 and 2 indicate mean lengths of reads*.

**Table 2:** cpDNA purity: coverage depths and copy ratio of plastid (pt), mitochondrial (mt), and nuclear (nc) genomes of the purified chloroplast DNA (cpDNA) and three total genomic DNAs.

	Depth			Copy ratio	
	pt	mt	nc	pt/nc	pt/mt
Purified cpDNA	3071	67	0.3	10172	45.7
Total DNA_1	668	44	2.2	301	15.3
Total DNA_2	1094	187	18.3	60	5.8
Total DNA_3	366	66	11.0	33	5.5

*cpDNA purity was calculated from coverage depth of each genome. After alignment on the plastid genome, unmapped reads were realigned on the mitochondrial genome, then the unmapped reads on the mitochondrial genome were realigned on the nuclear genome*.

**FIGURE 5:**
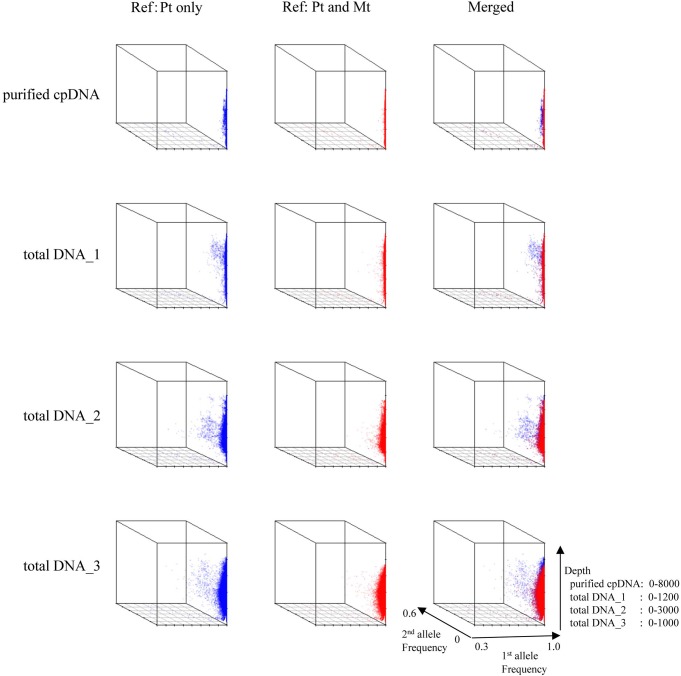
3D plots of allele frequency at individual base positions of the plastid genome of purified cpDNA and tDNA. Axes: *x*, first allele frequency; *y*, second allele frequency; *z*, coverage depth. Blue, plastid (Pt) reference genome; red, combined plastid–mitochondria (Mt) reference genome.

Next, we analyzed misaligned NUPT and MTPT reads, which disrupt precise analysis, on the plastid genome. We considered that MTPT reads containing a small number of mismatches against plastid genome could be separated by aligning reads to a combined plastid–mitochondria reference genome. Indeed, tDNA showed differences in coverage depth between the Pt and Pt–Mt reference genomes within regions of high similarity between the genomes, while purified cpDNA showed approximately similar coverage depth across the whole plastid genome (**Figure 4**; e.g., Region 1). To further detect NUPT- and MTPT-derived noise, we plotted the first and second allele frequencies and coverage depth at individual base positions on the plastid genome in 3D graphs (**Figure 5**). In the purified cpDNA, the first allele frequencies are close to 1.0 across the genome except in positions of low coverage depth, indicating low contamination by mitochondrial or nuclear DNA. By contrast, tDNA shows several sites where the first allele frequency was reduced and one or more other alleles were detected, even in positions with deep coverage depth. This result corroborates the frequency of this tendency and the lower purity of cpDNA (**Table 2**). Using the combined Pt–Mt reference genome improved the percentage of first allele frequencies. This result suggests that first allele reductions result from contamination by MTPTs, supporting observations of coverage depth differences such as in Region 1 in **Figure 4**. Furthermore, these results demonstrate that aligning on the combined Pt–Mt reference genome enables reduction of mtDNA contamination by a computational approach. However, it seems that the remaining low first allele frequencies are subject to noise derived from ncDNA. It is difficult to remove ncDNA-derived contamination by computation because some rice NUPTs have the same sequence as in the complete plastid reference genome and occur in multiple copies in the nuclear genome (Matsuo et al., 2005). The above results indicate that purified cpDNA can lead to high coverage depth of the chloroplast genome with a low number of reads, providing robust mapping and high-throughput sequencing of the rice plastid genome. Using tDNA sequenced on the Illumina platform is not consistently reliable, showing a higher rate of error alignment, which will likely affect later analysis such as plastid genome assembly or variant detection. It is important to highlight here the power of this technique in isolating cpDNA with improved data quality and lowered sequencing costs.

**FIGURE 6:**
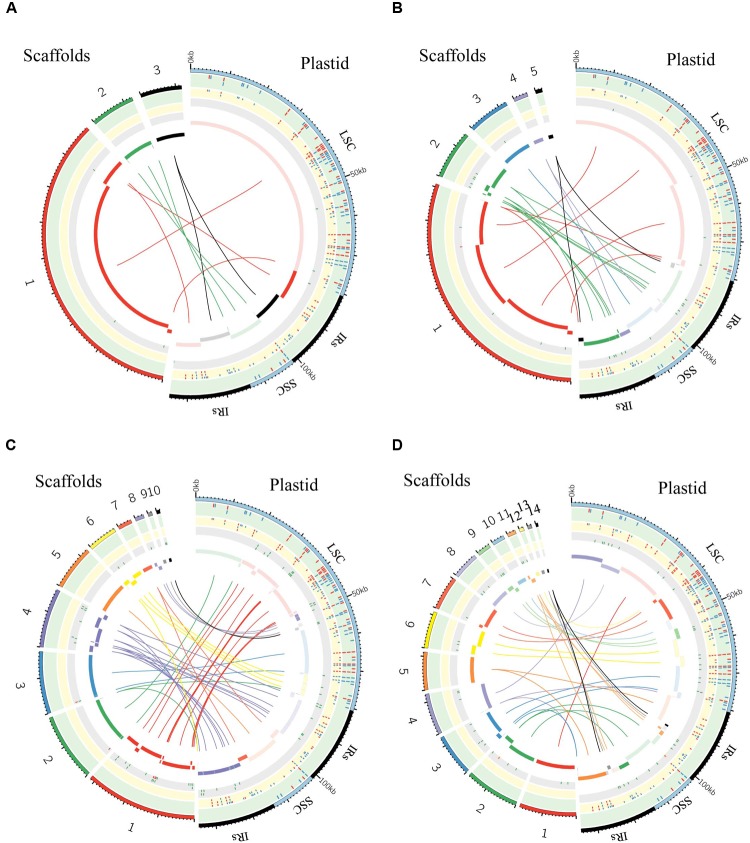
Graphic summary of *de novo* assembly of whole plastid genome. Scaffolds and SNPs/Indels positions: **(A)** purified chloroplast cpDNA; **(B)** the total genomic DNA_1; **(C)** the total genomic DNA_2; **(D)** the total genomic DNA_3. The assembled scaffolds (>500 bp) were aligned to the plastid reference genome by NCBI BLAST 2, and the hit regions are indicated by central colored ribbons and bars. Scaffold hits in the opposite direction to the reference genome are represented with lightest color in the bar on the side marked “Plastid.” Detected SNPs (red tick marks) and indels (blue tick marks) are plotted in the green (GATK, **Table 3**) and yellow (*de novo* assembly) tracks (Supplementary Table 3). Regions of undetermined base positions (‘N’; green tick marks) and sequencing artifacts such as insertions of mitochondrial homology sequence (red tick marks) and duplication of plastid sequence (orange tick marks) are plotted in the gray track (Supplementary Table 5). LSC, large single-copy; SSC, small single-copy; IRs, pair of inverted repeats.

The rice Nipponbare plastid reference genome X15901.1 was released in 1989 (Hiratsuka et al., 1989). Later, Tang et al. (2004) independently published a Nipponbare plastid reference genome, AY522330, noting 79 SNPs and 110 indels of putative sequence errors. To assess the inference of misaligned reads from the NUPTs and MTPTs by detection of these sequencing errors, we used AY522330 as correct data, and processed the BAM files describing the read alignments on X15901.1 to detect variants using two distinct variant callers, SAMtools mpileup and GATK HaplotypeCaller (**Table 3** and Supplementary Table 3). GATK HaplotypeCaller had much higher sensitivity than SAMtools mpileup, as previously reported (Liu et al., 2013; Pirooznia et al., 2014; Yi et al., 2014). SAMtools mpileup failed to detect most indels (Supplementary Table 3), having very low sensitivity in indel calling (Tian et al., 2016). As sample purity decreases, SAMtools detects more heterozygous variants, which may be false positives, as they don’t exist in AY522330. Although it is possible to select highly reliable variants by filtering out heterozygous and low-quality variants, some false-positive variants were found in all samples (e.g., plastid genome sites at 44772, 44775, 70290, and 70291; Supplementary Table 3). On the other hand, all variants identified by GATK HaplotypeCaller are consistent with AY522330 and show a high-quality score tolerant to the filtering process, regardless of sample purity, and no difference was found in the number of variants within the four samples. These results suggest that GATK HaplotypeCaller is superior at detecting mutations with high accuracy, not only in purified cpDNA, but also in total DNA samples, in the resequencing analysis of the plastid genome. However, GATK identified only 129 variants out of 189 reported in AY522330 (Tang et al., 2004). Most of those missing variants lie within the two IRs (**Figure 6** and Supplementary Table 3), since GATK HaplotypeCaller ignores low-mapping-score reads (e.g., multi-mapping reads). Similarly, population genetic analysis of chloroplasts in 383 rice varieties also showed lower SNP/indel density within the IRs than in other plastid genome regions (Tong et al., 2016). Our efforts to detect variants in the IRs by changing several parameters of GATK HaplotypeCaller to allow low-mapping-score reads did not resolve this issue (data not shown). Although GATK HaplotypeCaller is the current gold standard variant caller, our results show that the development of other computation approaches is required for plastome resequencing. The discrepancies between our purified cpDNA and total DNA studies shed light on the importance of plant plastidial studies to thoroughly describe how to map them.

**Table 3:** Plastid variant call results from SAMtools mpileup versus GATK HaplotypeCaller using the purified cpDNA and the three total genomic DNA samples.

	SAMtools mpileup				GATK HaplotypeCaller			
Aligned (ref)	Pt only		Pt and Mt		Pt only		Pt and Mt	
Filter	Total	Pass	Total	Pass	Total	Pass	Total	Pass
Purified cpDNA	86	64	86	64	129	129	129	129
Total DNA_1	86	66	86	67	129	129	129	129
Total DNA_2	131	64	87	65	129	129	129	129
Total DNA_3	128	69	99	69	129	129	129	129

*Processed reads were aligned on the plastid reference genome (Pt only) or on the combined plastid–mitochondrial reference genome (Pt and Mt). Heterozygous and low-quality variants (QUAL < 20) were filtered out in SAMtools, and low-quality variants (QUAL < 20) were filtered out in GATK*.

### Assessments of Plastome *de Novo* Assembly: Scaffolds and Variant Calling

We generated *de novo* assembled plastid genomes for assessment of the purified cpDNA and the three tDNAs (**Figure 6** and Supplementary Tables 3–5). Comparison of the scaffold alignments showed that the plastid genome was almost fully covered by the assemblies of all samples, but the tDNAs generated numerous shorter scaffolds. Repeat sequences are well known to hinder long contig formation (Kim et al., 2015). Our results also show that the contig elongations stop around the boundaries of the evolutionally conserved pair of large IRs. Since it is difficult to construct a complete plastid genome from a single library, supplementation with libraries of different insert sizes and mate pairs, long read sequencing, and PCR analysis is also necessary (Ferrarini et al., 2013; Naito et al., 2013).

The genome assembled *de novo* from the purified cpDNA has long contiguous sequences which were joined into three scaffolds in the correct order (**Figure 6A**). This is potentially another advantage of the LN approach. In contrast, *de novo* assembly using tDNA produced 5, 10, and 14 scaffolds (**Figures 6B–D**). A simulation study demonstrated that de Bruijn Graph Assemblers such as SOAPdenovo2, which we used, can improve the assembly of longer contigs from longer reads (Knudsen et al., 2010). Certainly, among tDNAs 2 and 3, the plastid DNA ratio was approximately the same, but the longer read length of tDNA 3 resulted in the assembly of longer contigs (**Figures 6C,D** and **Table 1**). Moreover, high heterozygosity increases the complexity of the de Bruijn graph structure, leading to small contigs and base call errors (Kajitani et al., 2014). Other reports also suggest that sequence error and GC bias create ‘dropouts’—multiple gaps in assemblies—and hence small contigs and scaffolds, even in small genomes such as those of plastids (Li et al., 2010; Minoche et al., 2011). In the *de novo* plastome assembly, widespread NUPTs and MTPTs are likely to behave like heterozygous sites and sequence errors, and thus to interrupt contig formation. In fact, our results show a clear tendency for scaffolds to be longer with purer plastid DNA (**Figure 6** and **Table 1**). Additionally, scaffolds in tDNAs contain multiple low-quality regions composed of both small and large gaps of consecutive undetermined (‘N’) bases and sequencing artifacts such as the insertion of regions with high homology to mitochondrial sequences and the duplication of plastid sequences (**Figure 6** and Supplementary Table 5). By contrast, we obtained scaffolds of purified cpDNA across the whole plastid genome with very few ‘N’ sequences.

As a result of SNP/indel detection, *de novo* sequencing identified 187 or 188 variants out of 189 reported in AY522330, with a higher sensitivity than resequencing analysis by GATK HaplotypeCaller (**Figure 6** and Supplementary Table 3). Despite this high sensitivity, tDNA 2 and 3 returned 41 and 58 additional variants, indicative of high false-positive rates, but the purified cpDNA did not return other variants. Taken together, these results reveal specificity and robustness in the identification of SNPs/indels by *de novo* sequencing with high-purity cpDNA.

Overall, read length and cpDNA purity are key to the successful *de novo* assembly of plastid genomes. Increasing cpDNA purity compensates for the low yield of MiSeq and enables the best use of its 300-bp paired-end sequencing, which is the longest read length of current Illumina next-generation sequencers. High-purity cpDNA is crucial for *de novo* assembly and SNP/indel calling without sequencing artifacts. It’s worth noting that *de novo* sequencing allowed the identification of SNPs/indels within the two IRs where GATK HaplotypeCaller ignored variant calling. This result indicates that *de novo* sequencing using high-purity cpDNA could be an effective method for detecting variants within IRs.

## Conclusion

The updated LN technique permits the extraction of enriched cpDNA, allowing the investigation of plastid genomes in a more cost-effective, time-saving manner, with huge increases in sequence throughput. We demonstrated that it is possible to obtain high-quality cpDNA with which to perform functional analysis to widen the scope for high-throughput sequencing and gain new insights for other genetic studies. Collectively, our analyses strongly support that the LN protocol increases the depth of coverage with a low output of short-read sequencing, allowing the large-scale bioinformatic/computational analysis of data. Using this protocol, we generated highly accurate plastid genome sequences without sequencing artifacts. The application of NGS followed by read mapping analysis to highly purified cpDNA would allow efficient detection of SNPs and indels within a plant population and accessions. This improvement in chloroplast sequencing technologies may help the rapid advancement of the chloroplast genomics field, the understanding of plastid genome replication and repair, the high-resolution analysis of heteroplasmy, and the development of technologies for chloroplast transformation.

## Accession Codes

The sequence data have been deposited in the DDBJ Sequence Read Archive: DRR118684.

## Author Contributions

TM and TK designed and supervised the research. TT performed the experiments and carried out the analysis. TI, KO, and TO contributed analytic tools. TT, MB, and TM interpreted the data. MB contributed to the conception and design of the work. TT, MB, and TM wrote the manuscript. MB, KI, and TM revised and finalized the paper. All authors read and approved the final version of the manuscript.

## Conflict of Interest Statement

The authors declare that the research was conducted in the absence of any commercial or financial relationships that could be construed as a potential conflict of interest.

## Footnotes

1 https://www.ncbi.nlm.nih.gov/genome/browse/?report=5

2 http://broadinstitute.github.io/picard/

3 https://github.com/linneas/condetri

## Abbreviations

BAM: binary alignment map
cpDNA: chloroplast DNA
HS: high salt method
InDel: small insertion/deletion
IRs: inverted repeats
LN: liquid nitrogen coupled with sucrose gradient centrifugation method
LSC: large single-copy region
Mt: mitochondria
MTPTs: mitochondrial plastid like sequences
NGS: next-generation sequencing
Pt: plastid
NUPTs: nuclear plastid DNA
PG: Percoll gradient centrifugation method
SNP: single-nucleotide polymorphism
SSC: single copy region.
